# Conversion of Waste Expanded Polystyrene into Blue-Emitting Polymer Film for Light-Emitting Diode Applications

**DOI:** 10.3390/polym15244693

**Published:** 2023-12-13

**Authors:** Huanyou Su, Hua Lin, Pengfei Li, Bowen Li, Xiaodong Xu, Jiacheng Li, Yuanquan Wu, Jiaqi Hui, Dan Liu

**Affiliations:** College of Biological, Chemical Sciences and Engineering, Jiaxing University, Jiaxing 314001, China

**Keywords:** waste expanded polystyrene, blue-emitting polymer film, light-emitting diode, benzimidazole group, chemical modification

## Abstract

The wide range of applications and continuous demand for plastics is causing serious global environmental problems. Massive discharges of expanded polystyrene (EPS) are thought to be primarily responsible for the increased white pollution. Waste EPS has received wide attention in the development of innovative products. White light-emitting diodes pumped by a near-UV chip (n-UV WLEDs) are regarded as a very promising solid-state lighting. The performance of the n-UV WLED is largely determined by the properties of the tricolor luminescence materials. In this work, a blue-emitting polymer film for n-UV WLED applications was developed from waste EPS. First, using waste EPS as a raw material, benzimidazole groups were bonded to PS benzene rings by chemical reactions to obtain modified PS (PS-PBI). Then, a film based on PS-PBI was prepared by a simple solution drop-casting method. The PS-PBI film can emit intense blue light when irradiated with 365 nm light. An n-UV WLED pumped by a 365 nm UV chip was fabricated using PS-PBI film as the blue-emitting layer. The fabricated n-UV WLED shows excellent luminescence properties, such as a bright white light with color coordinates of (0.337, 0.331), a relatively low color temperature (CCT, 5270 K), and an especially high color rendering index (CRI, 93.6). The results prove that the blue-emitting PS-PBI film prepared from waste EPS is a very promising candidate for n-UV WLED applications. The strategy of converting waste EPS into a high-value-added blue-emitting film in this work provides a convenient and feasible approach for upcycling waste EPS, achieving significant environmental and economic benefits.

## 1. Introduction

Plastics have been widely used in our daily lives due to their low cost, versatility, and durability. Global plastic demand has steadily increased over the years. Plastic production totaled 400.3 million tons in 2022 [1]. China was the largest annual producer in 2022, contributing to 32% of the total plastic production [1]. Plastic production is estimated to reach 1124 million tons in 2050 [2]. Extensive applications and the continuous demand for plastics have resulted in the accumulation of plastic waste, leading to increased environmental pollution [3,4]. According to estimates, 8300 million tons of plastic were produced between 1950 and 2015, of which approximately 6300 million tons were discarded as waste, while only 9% was recycled, 12% was incinerated, and the remaining plastic waste was either landfilled or disposed of in the natural environment [2]. The incineration of waste plastics can release harmful gases, while landfills or the direct disposal of waste plastics into nature will pose a serious threat to terrestrial and marine ecosystems. 

Polystyrene (PS) is one of the most commonly used thermoplastic, accounting for 6% of the world’s total plastic production [5]. PS is often used in the form of foams (known as expanded polystyrene (EPS)). EPS has been widely used as a packaging, building and construction, and insulation material because of its low weight, thermal insulation capacity, sound insulation, and excellent processibility [2,6,7]. However, most commercial EPS products are disposable. The massive quantity of discarded EPS is causing serious environmental problems. Landfilling and incineration are the most commonly used disposal methods of EPS waste. Compared to landfilling and incineration, recycling waste EPS into useful products is a more promising way to utilize waste EPS in terms of environmental protection and resource sustainability. Presently, the recycling methods for EPS waste mainly include dissolution recycling, melt treatment, mechanical recycling, high temperature pyrolysis, and chemical modification [8,9]. Chemical modification is an efficient technique for recycling waste EPS into various functional materials [10]. Different functional groups can be attached to PS benzene rings to confer certain functionalities and applications in various fields on PS by chemical reactions owing to the high reactivity of PS benzene rings. For example, sulfonic acid groups can be bonded to the PS benzene rings by a sulfonation reaction to achieve sulfonated PS, which has been found to have a wide range of applications in industrial wastewater as a polymeric flocculant [11,12,13,14]. Carboxyl groups can be attached to PS chains via the Friedel-Crafts reaction between PS and anhydrides to obtain carboxyl-functionalized PS, which can be used as an efficient adsorbent for removing cationic dyes and heavy metals in wastewater [15,16,17]. To develop innovative functional materials with added value from waste EPS by chemical modification is highly desirable for environmental protection and the efficient conversion of waste EPS.

Phosphor-converted white light-emitting diodes (pc-WLEDs) have been promised for the fourth generation of solid-state lighting (SSL) due to their high efficiency, long lifetime, low energy consumption and environmental friendliness [18,19,20]. Currently, the commercialized pc-WLEDs are commonly fabricated by integrating blue InGaN chips with yellow YAG:Ce^3+^ phosphors [21,22]. However, such WLEDs usually suffer from low color rendering index (CRI, <80) and high correlated color temperature (CCT, >7000 K) due to the lack of a red region [23,24]. Meanwhile, the strong blue emission from the blue InGaN chip causes irreversible damage to the human eye and several other health problems [25,26,27]. A promising alternative technology to overcome these issues is to employ near-ultraviolet (n-UV) (350–410 nm) chips to excite tricolor (e.g., blue/red/green) phosphors for developing the n-UV WLED devices [19,28,29]. The performance of the n-UV WLED devices are largely determined by the properties of tricolor phosphors [30,31]. Therefore, it is very critical to develop tricolor phosphors with excellent luminescence properties suitable for n-UV LED excitation. 

At present, among the available n-UV WLEDs tricolor phosphors, blue-emitting phosphors are limited [29,32]. Currently commercial blue-emitting phosphors are mostly rare earth inorganic phosphors. BaMgAl_10_O_17_:Eu^2+^ (BAM) is the most commonly used commercial blue-emitting phosphor [33,34]. However, such commercial blue-emitting phosphors often suffer from some disadvantages, such as high energy consumption due to high-temperature solid-state synthesis processes, expensive raw materials due to high-cost lanthanide ions, and in particular, poor compatibility when encapsulated in silica gel resins, which affect the luminescence stability of fabricated LED devices due to the aggregation of inorganic particles in the resins [19,33,35]. Using blue-emitting polymer-film-free lanthanide ions to directly paste n-UV chips onto fabric LED devices can overcome the above shortcomings of traditional blue-emitting inorganic phosphor. 

**Figure 1:**
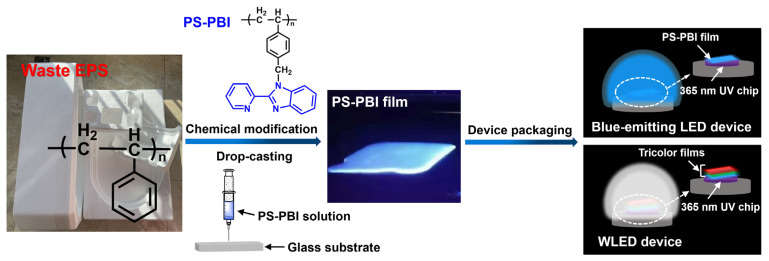
Schematic illustration of upcycling waste EPS into blue-emitting film for LED applications.

This work provides an approach for upcycling waste EPS into a value-added blue-emitting polymer film based on PS for n-UV WLED applications via chemical modification (Figure 1). Owing to the high reactivity of electron-rich benzene rings of PS, a blue-emitting polymer based on PS (PS-PBI) was first prepared from waste EPS by attaching benzimidazole groups to PS benzene rings by chemical reactions. Benzimidazole and its derivatives are considered classic blue emitters [36]. Then, a blue-emitting polymer film was prepared by a simple solution drop-casting method from the as-prepared PS-PBI due to the good solubility and casting ability of PS-PBI. Furthermore, the PS-PBI film was packaged on the 365 nm n-UV chip to make a blue LED device. Finally, an n-UV WLED with a high CRI (Ra = 93.6) was fabricated by packaging tricolor films, including the as-prepared blue-emitting PS-PBI film, as well as a red-emitting film (R-film) and a green-emitting film (G-film) based on red phosphor and green phosphor, as previously prepared in our group [37]. 

## 2. Materials and Methods

### 2.1. Materials

Waste EPS was collected from discarded polystyrene foam used for product packaging. Anhydrous tin chloride (SnCl_4_) and 2-(2-pyridyl) benzimidazole (PBI) were obtained from Aladdin Reagent (Shanghai, China). 1,4-bis(chloromethoxy)butane (BCMB) was purchased from Xi’an Lanjing Biotechnology Co., Ltd. (Xi’an, China). Dichloromethane (CH_2_Cl_2_); potassium hydroxide (KOH); carbon tetrachloride (CCl_4_); N,N-dimethylformamide (DMF); and acetonitrile (ACN) were purchased from Sinopharm Chemical Reagent Co., Ltd. (Shanghai, China). Industrial alcohol was supplied by Jiaxing Shengde Chemical Industry Co., Ltd. (Jiaxing, China). 

### 2.2. Recycling and Disposal of Waste EPS

The recycling and disposal of waste EPS was performed according to the method described in our previous report [37]. The discarded polystyrene foam was cut into slices. 2.0000 g slices dissolved in 40.00 mL CH_2_Cl_2_. The above solution was slowly poured into industrial alcohol. Then, the precipitate was filtered off and dried at 60 °C for 12 h in a vacuum oven to yield the purified PS. 

### 2.3. Preparation of PS-PBI 

0.2000 g of the purified PS was dissolved in 8.00 mL CCl_4_. 0.25 mL of SnCl_4_ was then added to the PS solution. 0.75 mL of BCMB was added to the above mixture solution and stirred continuously for 16 h at room temperature. The reaction solution was precipitated in industrial alcohol. The precipitate was filtered off and dried at 60 °C for 12 h in a vacuum oven to yield the chloromethyl polystyrene (CMPS). 

0.3852 g of PBI was dissolved in 4.00 mL of DMF. 0.3321 g of KOH was added to the PBI solution. A solution of 0.2000 g of CMPS in 4.00 mL of DMF was added to the above mixture solution and was heated to 70 °C and stirred for 10 h. The reaction was cooled to the room temperature and then precipitated in deionized water. The precipitate was washed with ACN three times, filtered off, and dried at 60 °C for 12 h in a vacuum oven to yield PS-PBI. 

### 2.4. Preparation of PS-PBI Film

0.3000 g of PS-PBI prepared was dissolved in 1.00 mL of DMF. The PS-PBI solution was deposited on a clean glass substrate by drop-casting and then dried at 60 °C for 12 h in a blast drying oven to yield PS-PBI film. 

### 2.5. Preparation of Blue-Emitting LED Device

A mixture of A and B glue (silicone resin) with the mass ratio of 1:4 was used to paste the as-prepared PS-PBI film of appropriate size on a commercial 365 nm UV chip, and it was dried at 100 °C for 2 h in a blast drying oven. Using this method, a blue-emitting LED device was prepared.

### 2.6. Preparation of WLED Device

To achieve a WLED device, the R-film and G-film were prepared by using the above purified PS, red phosphor (Eu^3+^ complex), and green phosphor (Tb^3+^ complex). The Eu^3+^ complex and Tb^3+^ complex were previously prepared by our group [37]. 

0.0010 g of the Eu^3+^ complex was dissolved in 0.30 mL of DMF. Then, the above solution was added to a solution of the purified PS (0.3000 g) in 1.00 mL of DMF. A uniform mixture solution was obtained under continuous stirring, which was deposited on a clean glass substrate by drop-casting, and then dried at 60 °C for 12 h in a blast drying oven to yield R-film. 

0.1000 g of the Tb^3+^ complex was dissolved in 0.30 mL of DMF. Then, the above solution was added to a solution of the purified PS (0.3000 g) in 1.00 mL of DMF. A uniform mixture solution was obtained under continuous stirring, which was deposited on a clean glass substrate by drop-casting, and then dried at 60 °C for 12 h in a blast drying oven to yield G-film. 

A WLED device was fabricated by packaging the as-prepared blue-emitting PS-PBI film, R-film, and G-film on a commercial 365 nm UV chip. The preparation process involved the tricolor films of suitable sizes first overlapping the chip. The mixture of A and B glue with the mass ratio of 1:4 was used as the adhesion agent between the two films or between the film and the chip. Then, the device was dried at 100 °C for 2 h in a blast drying oven to obtain the WLED device. 

### 2.7. Characterization

Gel permeation chromatography (GPC) analysis was conducted using an HLC-8320GPC (TOSOH, Japan) equipped with a TSKgel GMHXL column using tetrahydrofuran (THF) as an eluent with a flow rate of 1 mL/min at 40 °C. Fourier transform infrared spectra (FTIR) were obtained using a Nexus 470 FT-IR spectrophotometer (Thermo Nicolet, USA) in the 4000–400 cm^−1^ region using KBr pellets at room temperature. Nuclear magnetic resonance spectra (^1^H NMR) were obtained in deuterated dimethyl sulfoxide (CHCl_3_-d_6_) using a 400M spectrometer (Varian, USA) with tetramethylsilane (TMS) as the internal reference. The excitation and emission spectra of the PS-PBI solution were measured on a Cary Eclipse fluorescence spectrophotometer (Agilent, USA), while the excitation and emission spectra of the PS-PBI film were measured using an F-4600 fluorescence spectrophotometer (Hitachi, Japan), and the excitation and emission spectra of the R-film and G-film were measured using a FS5 steady-state and time-resolved photoluminescence spectrometer (Edinburgh, UK). Digital images were taken using a Coolpix P7000 camera (Nikon, Japan). The properties of the LEDs were characterized using a HP9000 spectrophotocolorimeter (Hongpu Optoelectronic Technology Co., Ltd., China). 

## 3. Results and Discussion

### 3.1. Purification of Waste EPS

**Figure 2:**
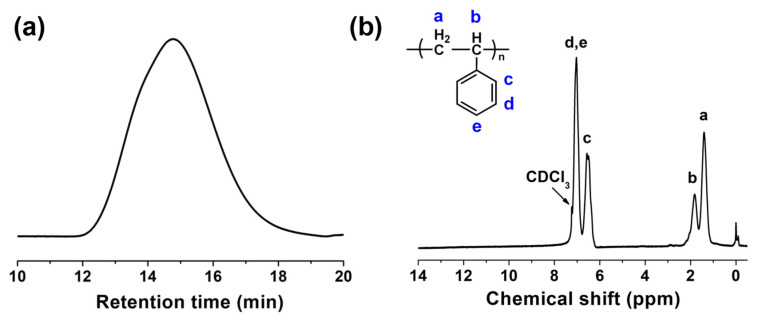
PS: (**a**) GPC curve, (**b**) ^1^H NMR spectrum.

The collected discarded polystyrene foam was purified by a dissolution and precipitation process. That is, the discared foam packaging was cut into slices, then dissolved in CH_2_Cl_2_ and precipitated in industrial alcohol to obtain purified PS. Figure 2a shows the GPC results of the purified PS. It can be seen that the purified PS has a number average molecular weight (M_n_) of 94,863, weight average molecular weight (M_w_) of 230,223, and dispersity (M_w_/M_n_) of 2.43. The structure of the purified PS was further characterized by ^1^H NMR measurement, and the result is shown in Figure 2b. As shown in Figure 2b, the peaks of 7.03 ppm and 6.56 ppm correspond to the proton chemical shifts of the benzene rings of PS, and the peaks of 1.82 ppm and 1.41 ppm are attributed to the proton chemical shifts of the olefin skeleton of PS. The above results suggest that pure PS was obtained by a simple dissolution and precipitation process from waste EPS.

### 3.2. Preparation and Characterization of PS-PBI

**Figure 3:**
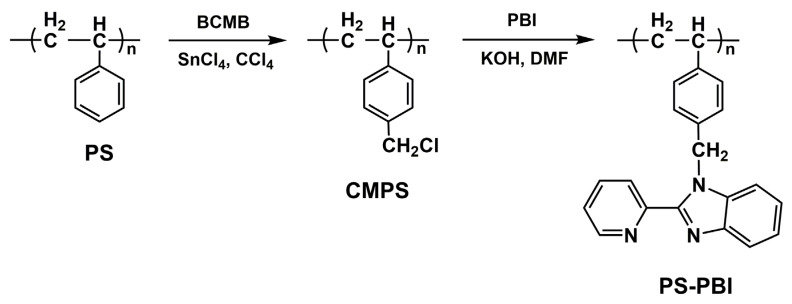
Synthesis route of PS-PBI.

**Figure 4:**
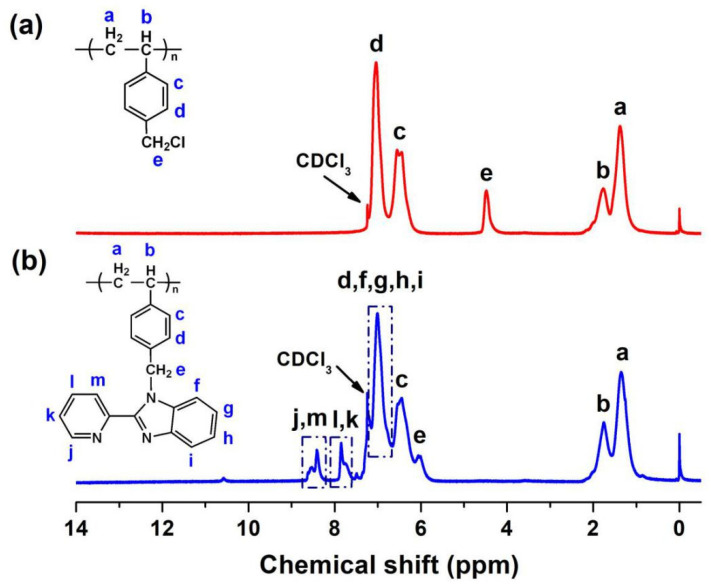
^1^H NMR spectra of CMPS (**a**) and PS-PBI (**b**).

Using the purified PS obtained as raw materials, PS-PBI was synthesized by chemical reactions, and the synthesis route is shown in Figure 3. Chloromethyl groups were first attached to the para positions of the PS benzene rings using BCMB as the chloromethyl reagent to yield CMPS. Then, the benzimidazole groups were introduced into the side chain of PS by the reaction between the chloromethyl groups of CMPS and the active hydrogens of PBI to yield PS-PBI. The structure of PS-PBI was characterized by the ^1^H NMR spectrum, as shown in Figure 4, in which the ^1^H NMR spectrum of CMPS is also given for comparative analysis. A new peak of 4.48 ppm can be observed in the ^1^H NMR spectrum of CMPS in Figure 4a compared to that of PS in Figure 2b, which can be assigned to the chemical shift of chloromethyl group protons on benzene rings. However, this peak is absent in the ^1^H NMR spectrum of PS-PBI, as shown in Figure 4b, while a peak at 6.06 ppm corresponding to the chemical shift of methylene protons appears [38]. Meanwhile, the new peaks between 7.70 ppm and 8.60 ppm assigned to the pyridine ring protons are observed [39,40]. In addition, the peak at about 7.00 ppm attributed to the benzene ring protons becomes broad due to the introduction of the benzene rings from the benzimidazole groups in the PS-PBI. The attribution of the peaks of CMPS and PS-PBI can be seen in detail in Figure 4, respectively. The results prove that PS-PBI was successfully synthesized from waste EPS.

### 3.3. Luminescence Properties of PS-PBI

**Figure 5:**
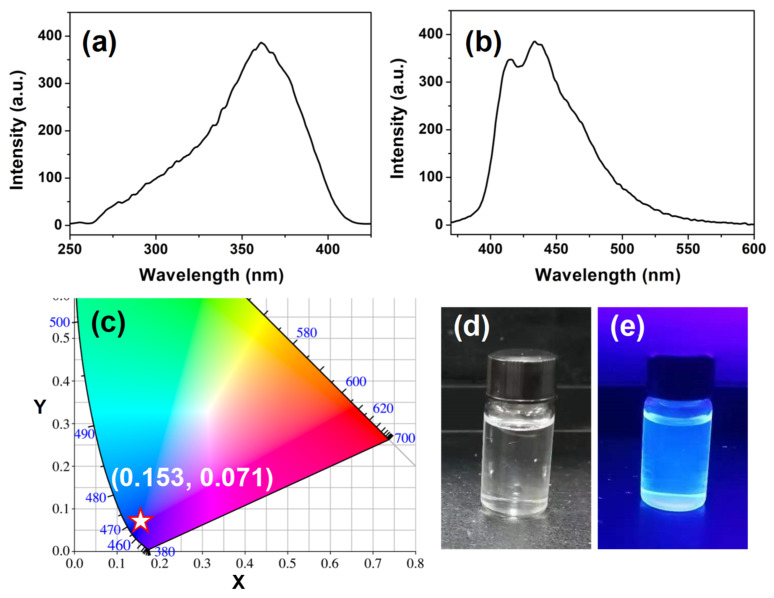
PS-PBI solution: (**a**) excitation spectrum, (**b**) emission spectrum, (**c**) CIE 1931 chromaticity diagram, (**d**) under daylight, (**e**) under 365 nm light.

**Figure 6:**
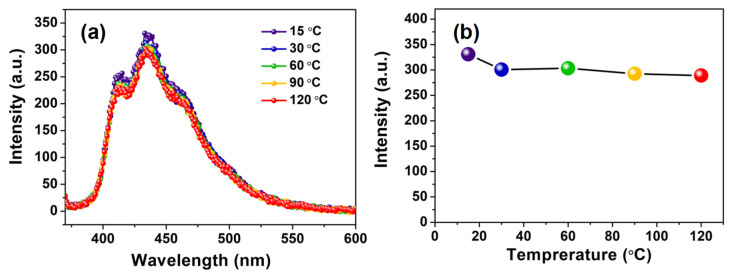
PS-PBI solution: (**a**) temperature-dependent emission spectra, (**b**) curve of relative emission intensity versus temperature.

To study the luminescence properties of the as-prepared PS-PBI, the excitation and emission spectra of the PS-PBI solution were investigated and are shown in Figure 5. The concentration of the PS-PBI solution in DMF is 1 × 10^−5^ mol/L. As illustrated as in Figure 5a, PS-PBI exhibits a broad excitation band centered at 360 nm in the range of 280 to 400 nm, which is attributed to the π→π* electronic transitions of the conjugated aromatic rings of PS-PBI, especially the benzimidazole groups [41,42]. Under 360 nm excitation, PS-PBI emits intense luminescence in the range of 400 to 450 nm (Figure 5b). The strongest peak is located at 433 nm, corresponding to blue light owing to the blue-emitting benzimidazole groups bonded to PS [41,43]. The CIE coordinate calculated according to the emission spectrum (Figure 5b) is (0.153, 0.071), which falls in the blue region in the CIE chromaticity diagram (Figure 5c). The PS-PBI solution is uniform and transparent under daylight (Figure 5d), suggesting that PS-PBI has excellent solubility in DMF. Under 365 nm UV light, the intense blue light emitted from the PS-PBI solution can be readily observed by the naked eye (Figure 5e), which is consistent with the result of the corresponding emission spectrum in Figure 5b. Temperature-dependent emission spectra of the PS-PBI solution excited at 360 nm were further investigated in the temperature range from 15 °C to 120 °C. The results are presented in Figure 6. As can be seen from Figure 6a, the emission peak positions of the PS-PBI solution under all temperatures remain unchanged, and the strongest peaks are all located at 433 nm. The relative emission intensities of the PS-PBI solution with the increase in temperature decrease only slightly, which can be seen more clearly from the curve of the relative emission intensity at 433 nm versus the temperature shown in Figure 6b. These results verify that a UV-excited blue-emitting PS-PBI was successfully prepared from waste EPS by chemical modification. Moreover, the blue-emitting intensity of the PS-PBI solution has a small thermal quenching property, which is beneficial for LED applications.

### 3.4. Luminescence Properties of PS-PBI Film and Its Application in LED Devices

**Figure 7:**
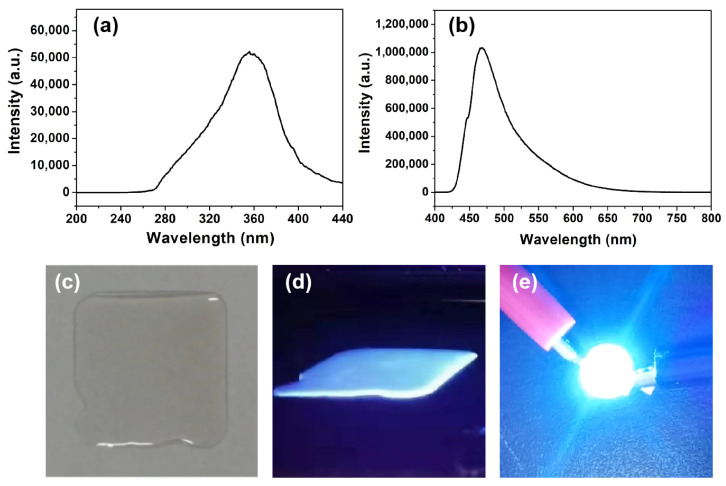
PS-PBI film: (**a**) excitation spectrum, (**b**) emission spectrum, (**c**) under daylight, (**d**) under 365 nm light, (**e**) LED device pumped by 365 nm UV chip.

The PS-PBI film was obtained via a simple solution drop-casting method from the as-prepared PS-PBI (Figure 1). The luminescence properties the of the PS-PBI film were investigated, and its excitation and emission spectra are shown in Figure 7a and Figure 7b, respectively. The PS-PBI film shows a broad excitation band in the 300 to 400 nm range in the n-UV region (Figure 7a). The maximum excitation peak is 356 nm, which is close to 365 nm. The results indicate that the PS-PBI film is suitable for LED devices pumped by an n-UV LED, especially by a 365 nm n-UV chip. Upon excitation at 356 nm, the PS-PBI film exhibits a strong emission band centered at 468 nm corresponding to blue light (Figure 7b). As illustrated in Figure 7c, the PS-PBI film possesses a smooth and uniform surface mainly due to good film-formation property of the PS-PBI. Not surprisingly, it emits intense blue light under 365 nm light (Figure 7d). An LED device was further fabricated by pasting the PS-PBI film on a commercial 365 nm UV chip, as shown in Figure 7e, which emits bright blue light when powered on. This proves that the LED device with strong blue light can be obtained using the PS-PBI film prepared from waste EPS as the emitting layer, suggesting that the PS-PBI film is an enticing luminescence material for LED applications.

**Figure 8:**
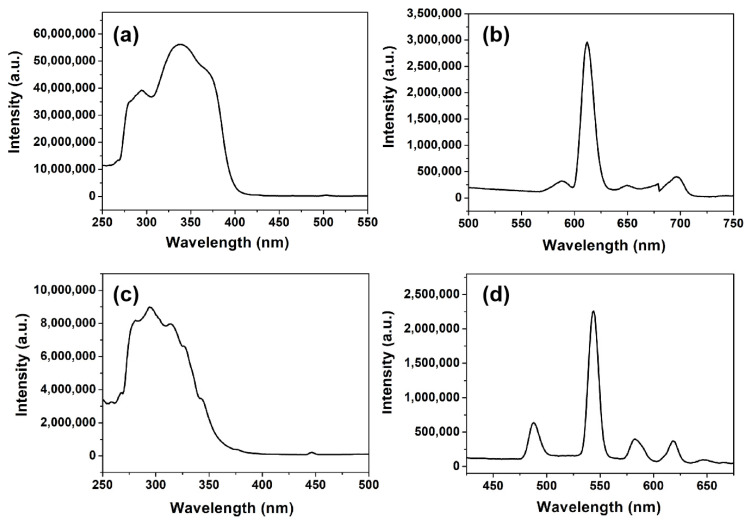
R-film: (**a**) excitation spectrum, (**b**) emission spectrum. G-film: (**c**) excitation spectrum, (**d**) emission spectrum.

**Figure 9:**
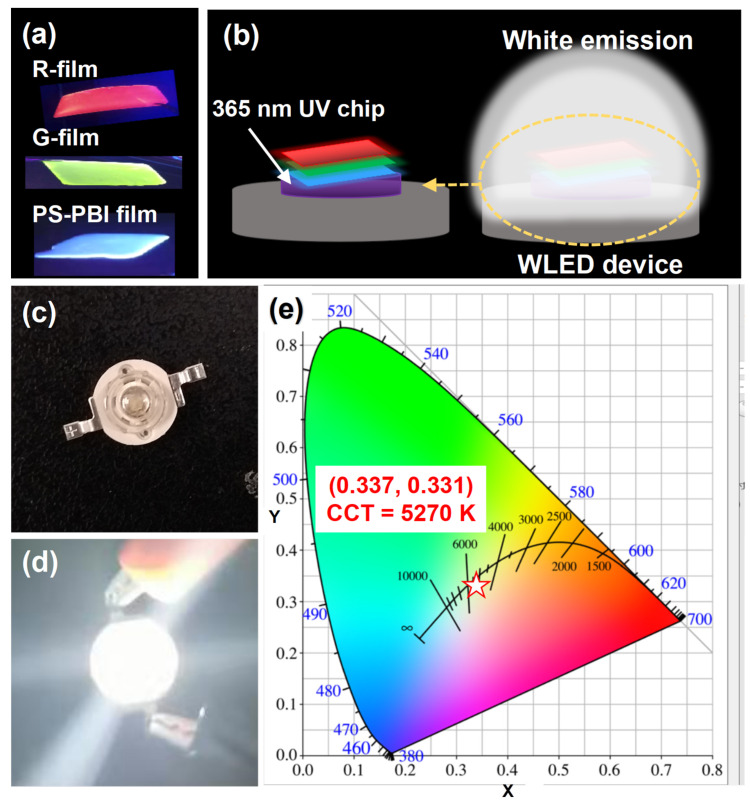
(**a**) R-film, G-film, and PS-PBI film under 365 nm light, respectively. WLED based on the tricolor films: (**b**) structural diagram, (**c**) not powered, (**d**) powered on, (**e**) CIE 1931 chromaticity diagram.

Taking advantage of the strong blue emission of the PS-PBI film, a WLED device pumped by a commercial 365 nm UV chip was fabricated by combining the PS-PBI film with R-film and G-film. Herein, the R-film and G-film were prepared from the Eu^3+^ complex, Tb^3+^ complex, and purified PS by a solution mixture and drop-casting method, respectively. Notably, PS is usually used as polymer matrix for luminescence composite films owing to its low cost, easy processing, and high transparency [35]. Figure 8 shows the excitation and emission spectra of the R-film and G-film, respectively. Both the R-film and G-film display broad excitation bands in the UV region, which can be attributed to the electronic transitions of the ligands in the complexes. Their maximum excitation peaks are 339 nm and 295 nm, respectively. The strong characteristic emission peaks of the Eu^3+^ ion and Tb^3+^ ion can be observed in the corresponding emission spectra of the R-film and G-film, respectively. Their maximum emission peaks at 612 nm and 544 nm are attributed to the ^5^D_4_→^7^F_5_ transition of the Tb^3+^ ion and the ^5^D_0_→^7^F_2_ transition of the Eu^3+^ ion, corresponding to red light and green light, respectively. As illustrated in Figure 9a, the tricolor films are capable of emitting bright red, green, and blue light under 365 nm light, respectively, suggesting that these films are suitable for LED devices pumped by a 365 nm UV chip. As depicted in Figure 9b, the tricolor films were stacked and adhered to a commercial 365 nm UV LED chip to manufacture the WLED device (Figure 9c). The as-fabricated WLED device emits intense white light when powered on (Figure 9d). The measured color coordinate is (0.337, 0.331), close to that of pure white light [44], which is located in the white region in CIE 1931 chromaticity diagram (Figure 9e). The measured CCT of the device is 5270 K, corresponding to neutral white light. The CRI of the WLED device was as high as 93.6, which is higher than that of the previously reported n-UV WLEDs [19,29,45,46]. Meanwhile, compared to commercialized pc-WLEDs fabricated by combining a blue InGaN chip with yellow YAG:Ce^3+^ phosphor [45,47,48], the n-UV WLED here exhibits a relatively lower CCT and higher CRI. These findings confirm that the PS-PBI film prepared from waste EPS could be a promising candidate for blue-emitting materials for fabricating n-UV WLEDs with excellent luminescence quality, particularly high CRI.

## 4. Conclusions

In conclusion, an effective method for converting waste EPS into high-value-added blue-emitting PS-PBI film for n-UV LED applications has been successfully developed. Due to the high reactivity of PS benzene rings, the benzimidazole groups were first attached to the benzene rings of PS chains via chemical reactions to achieve PS-PBI. Subsequently, the blue-emitting PS-PBI film was prepared based on the excellent solubility and film-formation properties of PS-PBI by a simple solution drop-casting method. The PS-PBI film with the maximum excitation peak of 356 nm can match the commercial 365 nm UV chip. The LED device with bright blue light was successfully fabricated by stacking the PS-PBI film on a 365 nm UV chip. Furthermore, the n-UV WLED device with intense white emission with a lower CCT of 5270 K, and particularly, a higher CRI (93.6), was achieved by combining the PS-PBI film with the R-film and the B-film. These results prove that the blue-emitting PS-PBI film prepared from waste EPS in this work is highly promising for n-UV WLED applications. Therefore, this work is of great significance for environmental remediation and the upcycling of waste plastics.

## Data Availability

Data are contained within the article.
